# Impact of coronavirus disease 2019 on healthcare workers: beyond the risk of exposure

**DOI:** 10.1136/postgradmedj-2020-137988

**Published:** 2020-06-19

**Authors:** Dimitrios Giannis, Georgios Geropoulos, Evangelia Matenoglou, Demetrios Moris

**Affiliations:** Institute of Health Innovations and Outcomes Research, Northwell Health Feinstein Institutes for Medical Research, Manhasset, New York, USA; Thoracic Surgery Department, University College London Hospitals NHS Foundation Trust, London, UK; Medical School, Aristotle University of Thessaloniki, Aristotle University of Thessaloniki, Thessaloniki, Greece; Duke Surgery, Duke University Medical Center, Durham, North Carolina, USA

In December 2019, a previously unknown coronavirus strain disease, the coronavirus disease 2019 (COVID-2019) or severe acute respiratory syndrome coronavirus 2 (SARS-CoV-2), emerged in Wuhan, China, and has rapidly spread worldwide.^1  2^ As of 5 April 2020, more than 1 000 000 people have been officially diagnosed and over 60 000 patients have died, while the pandemic is still spreading.^3^ Clinical manifestations range from asymptomatic or very mild to severe illness, sepsis and death. While information so far suggests that most COVID-19 illness is mild, severe illness occurs in up to 16% of cases.^4  5^ The clinical presentation is occasionally atypical, but patients usually present with fever (>80%), cough (>60%) and myalgia or fatigue (>40%).^1  4^ COVID-19 has been associated with high risk of acute respiratory distress syndrome and intensive care unit (ICU) admission.^1  2  6^ Currently, it is hard to predict the long-term impact of the pandemic on healthcare facilities and personnel. Healthcare workers, being in the frontline of an ongoing war against the pandemic, should be perceived as a discrete population in terms of both physical and mental health impact.

During a crisis, similar to the COVID-19 pandemic, shortages of drug and life-saving equipment may occur.^7  8^ COVID-19 has overwhelmed the capacity of healthcare resources and has significantly changed the workplace rules of healthcare workers.^9  10^ It has been recognised that healthcare workers should take appropriate precautions to avoid contracting the disease and prevent spread within the hospital. However, during the early stages of the pandemic, the lack of knowledge resulted in high rates of transmission of COVID-19 to healthcare workers, due to inadequate protection.^11  12^ Currently, the unprecedented overwhelming demand of protective equipment, which includes masks, medical gowns, gloves and eye–face protective devices, poses a significant health risk. Contracting the infection results in missing workdays, due to placement on quarantine, and increases the risk of disease transmission to family members. If the healthcare worker becomes severely affected, the need for hospitalisation and/or ICU admission emerges. The combination of increased workload, personnel shortage, risk of transmission and lack of resources severely affects the physical and mental health of healthcare workers and places healthcare systems under extreme burden.

This stressful situation and accumulated fatigue severely affect many aspects of work and personal life. Higher workload results in limited time for self-care, relaxation and even fulfilment of basic needs, including nutrition and self-hygiene.^13^ Social interaction is limited, while the application of social distancing in the healthcare workers’ population is difficult. Clinic rounds, interactive cases’ discussion, work-breaks for lunch occur within closed spaces and distancing is not always feasible.^10^ Isolation and self-neglect may eventually result in anger, irritability and mood instability. Further, the daily contact with patients and the scarcity of resources are factors that contribute to the overall stress that healthcare workers undergo during this time.

One of the most important issues is that healthcare practitioners may occasionally be confronted with ethical dilemmas of prioritising patients, based on risk factors, disease severity and resources availability. Ethical dilemmas and constant exposure may result in detrimental effects, both short- and long-term, in the mental health and well-being of this population. Kang and colleagues estimated the impact of the COVID-19 pandemic in the mental health of physicians and nurses in Wuhan, soon after the onset of the pandemic. Interestingly, half of the healthcare population had received psychological support through materials available online or provided by media, one out of three had obtained paper-based psychological counselling (brochures, leaflets or books), while approximately one out of five had received individual or group psychotherapy. Further, higher levels of healthcare workers’ distress were associated with the degree of exposure to infected patients.^14^ Previously, during the severe acute respiratory syndrome coronavirus 1 (SARS-CoV-1) 2003 outbreak, Wu *et al* reported a 10% frequency of post-traumatic stress (PTS) in hospital employees. Those who had been placed in quarantine, worked in high-risk facilities or had close contacts (friends or family members) affected by SARS-CoV-1 were at up to threefold higher risk of having severe PTS symptoms.^15^ Similarly, Chong and colleagues reported a 75.3% overall prevalence of psychiatric manifestations (anxiety, depression, sleep disturbances) in a population of healthcare workers employed in a tertiary hospital during the SARS-CoV-1 outbreak. The frequency was even higher during the subsiding/control phase (80.6%) compared with the early phase (71.3%) of the epidemic.^16^

The psychological stress imposed on healthcare practitioners varies and depends on physician expertise and practice site. As the pandemic continues to rapidly spread throughout the USA, one of the most severely affected physician groups are primary care doctors. Significant changes have occurred over a short period. Lack of official guidance and absence of a unified healthcare system that currently consists of small private practices and parts of community, university, federal or private hospital systems significantly hinder the rapid and effective application of proposed changes and regulations.^17^ Constantly changing recommendations on testing and patients’ triage mostly affect small private practices, that suffer from lack of protective resources and are financially dependent on patients’ visits, without any guarantee of reimbursement for telemedicine appointments.^17^ Recently, Greenhalgh and colleagues published a comprehensive guide about remote assessments in primary care to help primary care physicians to deal with telephone- or video-based conversations with patients.^18^

The effective management of emerging issues depends on the successful collaboration between interested parts. Hospital committees, stakeholders and federal agencies should make sure that adequate protective devices and tools become available as soon as possible to healthcare workers. In China, a monitoring system of exposed healthcare personnel has been used to facilitate early detection, appropriate triage and prompt isolation of healthcare personnel.^19^ This approach should be taken cautiously, and issues regarding privacy and breach of confidentiality should be resolved prior to widespread implementation. In the USA, volunteer programmes have been established and are expected to significantly contribute to the relief of overwhelmed hospitals.^20  21^ In the presence of volunteers, physicians may need to slightly deviate from the established guidelines and many of them will need time to appropriately practice beyond their expertise areas. Notably, retired healthcare workers and recent graduates will need extensive training to practise medicine safely.

Proposed measures to counteract the effect of COVID-19 pandemic on the mental health of healthcare workers include multidisciplinary psychiatric support teams, up-to-date information dissemination to relieve anxiety and uncertainty, psychological support through electronic devices (mobile phones), mental health screening and early, appropriate treatment in those affected by severe psychiatric symptoms.^22^ In addition, proper clinical recommendations for the management of patients treated for other diseases are of great importance, because healthcare workers are stressed by the possibility of disease transmission that may occur during rounds and between wards. Anelli and colleagues proposed that rapid tests, which provide results within 15–45 min, should be given to healthcare workers suspected of having COVID-19, even if they are experiencing only mild symptoms.^23^ To our perspective, this approach could significantly decrease the stress and doubts of healthcare workers that derive from the risk of potential transmission to close contacts and patients.

Institutional agencies and supervisors should be able to recognise the detrimental effects of the pandemic on healthcare workers and should be willing to decrease working hours, apply flexible schedules and clearly assign roles and responsibilities to equally distribute the workload.^13  24^ Extended work shifts can potentially affect the overall health and predispose to higher risk of acquiring respiratory infections.^25  26^ Recent reports raised awareness about skin disease, including abrasions and ulcerations, on the hands and face of healthcare workers due to the prolonged or repeated use of gloves/antiseptics or facial goggles/N95 mask, respectively.^27^ Therefore, staff should be given the opportunity to discuss decisions regarding their tasks and the equipment they use, and should regularly be evaluated on their well-being. Healthcare managers should promote adequate sleep, hygiene and take initiatives to supply healthcare workers with water, food and short break intervals.^28^ In addition, authorities, legislators and government agencies should show support and empathy in the event of adverse outcomes.^29^ Once the pandemic is over, affected and/or involved healthcare workers must be followed-up, supported, and long-term consequences should be appropriately treated.^30^

Academic societies and scientific associations have published guidelines to deal with the emerging issues in populations hospitalised for other diseases.^11^  ^31–33^ Guidelines, during this unprecedented event can significantly decrease the stress of dilemmas regarding the management of the elective, acutely ill and terminally-ill hospitalised population.

Lastly, as a society, we owe a clear and honest ‘Thank you for taking care of people’ to the healthcare workers’ population. As Bergman and colleagues state, what we need is physical distancing with social connectedness. This pandemic seems as a chance to reconsider and reinvest in relationships.^34^ No formalities, no excuses, no well-spoken politics. All they need is a ‘thank you’, from the bottom of our hearts, every time they sacrifice themselves to save people. Our people.
